# Supplementary material for: The causal relationship between the human gut microbiota and pyogenic arthritis: a Mendelian randomization study
Source: Front Cell Infect Microbiol. 2024 Nov 26;14:1452480. doi: 10.3389/fcimb.2024.1452480 (PMC11629706; doi:10.3389/fcimb.2024.1452480)
Supplement: Supplementary file 1 [file DataSheet1.zip › mendelian test/ebi-a-GCST90027491.csv_forest.pdf]

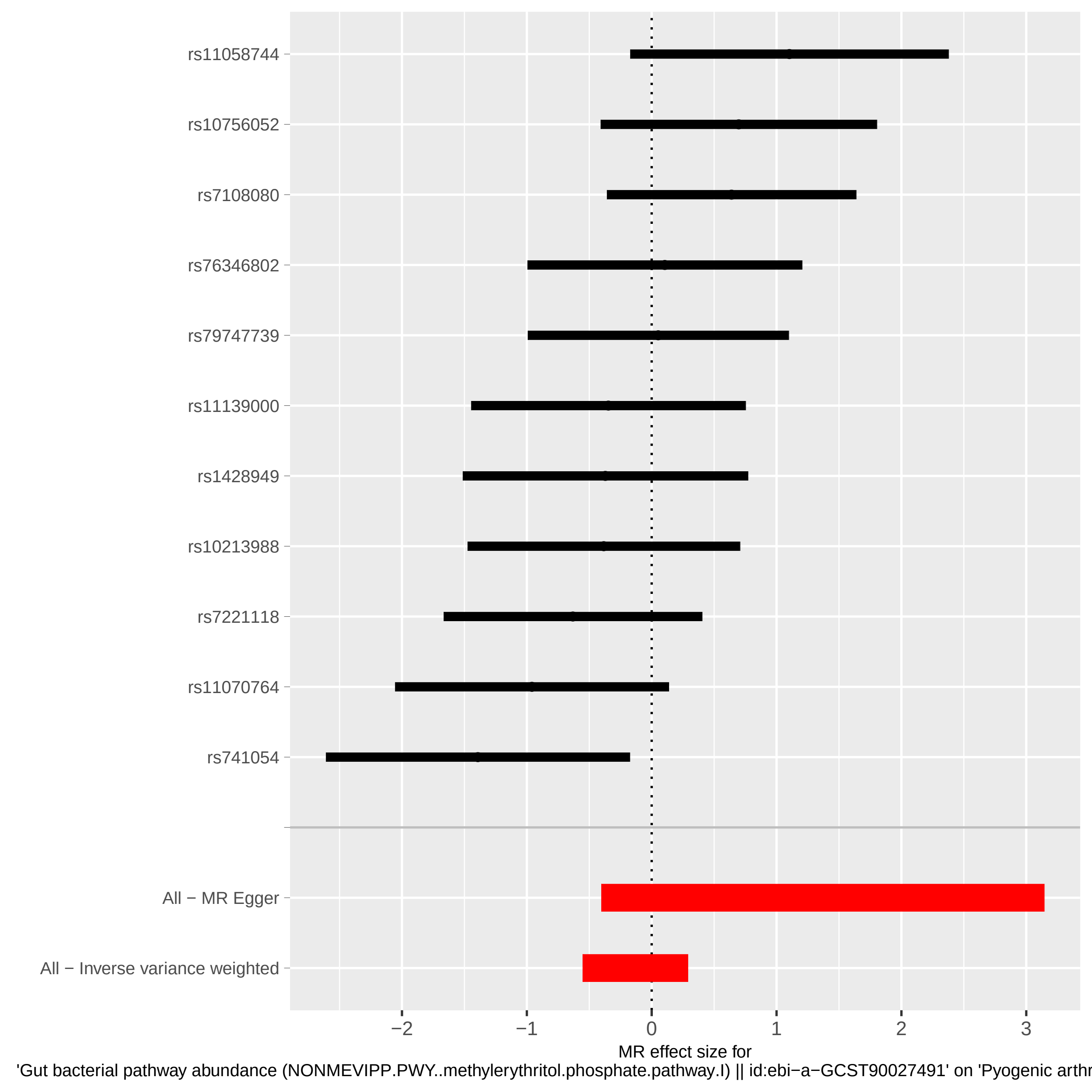

rs11058744
rs10756052
rs7108080
rs76346802
rs79747739
rs11139000
rs1428949
rs10213988
rs7221118
rs11070764
rs741054
All − MR Egger
All − Inverse variance weighted
−2
−1
0
1
2
3
MR effect size for
'Gut bacterial pathway abundance (NONMEVIPP.PWY..methylerythritol.phosphate.pathway.I) || id:ebi−a−GCST90027491' on 'Pyogenic arthr
